# Predictors of Post-Exercise Energy Intake in Adolescents Ranging in Weight Status from Overweight to Severe Obesity

**DOI:** 10.3390/nu14010223

**Published:** 2022-01-05

**Authors:** Nicole Fearnbach, Amanda E. Staiano, Neil M. Johannsen, Daniel S. Hsia, Robbie A. Beyl, Owen T. Carmichael, Corby K. Martin

**Affiliations:** 1 Pennington Biomedical Research Center, Baton Rouge, LA 70808, USA; amanda.staiano@pbrc.edu (A.E.S.); neil.johannsen@pbrc.edu (N.M.J.); Daniel.Hsia@pbrc.edu (D.S.H.); Robbie.Beyl@pbrc.edu (R.A.B.); owen.carmichael@pbrc.edu (O.T.C.); Corby.Martin@pbrc.edu (C.K.M.); 2 School of Kinesiology, Louisiana State University, Baton Rouge, LA 70803, USA

**Keywords:** appetite, prospective food consumption, uncontrolled eating, childhood obesity, weight gain

## Abstract

Exercise may sensitize individuals with overweight and obesity to appetitive signals (e.g., hunger and fullness cues), overriding trait eating behaviors that contribute to overeating and obesity, such as uncontrolled eating. The objective of the current study was to measure predictors of objective ad libitum energy intake at a laboratory-based, post-exercise test-meal in adolescents ranging in weight status from overweight to severe obesity. We hypothesized that appetitive states, rather than appetitive traits, would be the strongest predictors of energy intake at a post-exercise test-meal, after controlling for body size. At Baseline, 30 adolescents (ages 10–16 years, 50% female (F), 43% non-Hispanic white (NHW), 83% with obesity (OB)) completed state and trait appetite measures and an ad libitum dinner meal following intensive exercise. Nineteen of those participants (47% F, 32% NHW, 79% OB) completed identical assessments two years later (Year 2). Energy intake (kcal) at each time point was adjusted for fat-free mass index (i.e., body size). Adjusted energy intake was reliable from Baseline to Year 2 (ICC = 0.84). Multiple pre-meal appetite ratings were associated with test-meal energy intake. In stepwise linear regression models, pre-meal prospective food consumption was the strongest and only significant predictor of test-meal energy intake at both Baseline (R^2^ = 0.25, *p* = 0.005) and Year 2 (R^2^ = 0.41, *p* = 0.003). Baseline post-exercise energy intake was associated with weight change over two years (R^2^ = 0.24, *p* = 0.04), but not with change in fat mass (*p* = 0.11). Appetitive traits were not associated with weight or body composition change (*p* > 0.22). State appetite cues were the strongest predictors of post-exercise energy intake, independent of body size. Future studies should examine whether long-term exercise programs enhance responsiveness to homeostatic appetite signals in youth with overweight and obesity, with a goal to reduce excess energy intake and risk for weight gain over time.

## 1. Introduction

Studies in youth with obesity suggest that there are trait-level differences in eating behavior (i.e., behaviors that are stable over time) compared to healthy weight youth, leading to a vicious cycle of overeating and obesity [1,2,3]. Commonly hypothesized contributors to overeating include disinhibition and/or impulsivity, which reflect an inability to stop the cognitive or behavioral response to food stimuli [4,5,6,7,8]. These traits are often described as an unintentional tendency to overeat in the presence of food due to the inability to stop or limited cues to stop eating. Additional appetitive traits that are commonly implicated in obesity include cognitive restraint, emotional eating, and food motivation or food-related reward responsiveness [9,10,11,12]. Extremes in these types of eating behavior traits can be maladaptive, leading to sustained positive energy balance and weight gain over time [9,11,13,14].

Beyond trait-level eating behaviors, individual differences in appetitive states (e.g., factors such as hunger, fullness, prospective food consumption) and sensitivity to fluctuations in appetite can influence food-seeking behavior and energy intake [15,16]. Appetitive states fluctuate throughout the day based on energy status (fed vs. fasted), circadian rhythms, typical meal patterns, and other environmental factors [17,18]. Less is known regarding the role of appetitive states in weight gain and obesity, but some studies have suggested that people who are less responsive to satiety cues are at a greater risk [19,20,21,22]. In individuals who are predominantly sedentary, exercise is thought to help sensitize them to hunger and satiety signals. This results in people being responsive to both homeostatic hunger and satiety signals, while minimizing eating that is driven by the pleasurable aspects of foods or other environmental stimuli. Classic studies have shown that a sedentary lifestyle is associated with overconsumption, while an active lifestyle puts daily energy intake back in line with energy expenditure [23,24]. Therefore, exercise may sensitize individuals with overweight and obesity to homeostatic appetitive signals, more closely linking them to homeostatic energy needs.

Energy needs vary based on body size, growth, and physical activity levels, and are common drivers of energy intake. Fat-free mass is an indicator of body size and a primary determinant of resting metabolic rate and total energy expenditure, therefore contributing significantly to daily energy needs. Several studies have documented fat-free mass as a predictor of meal size, single meal food intake, and energy intake over the course of a day in both adults and children [25,26,27,28,29,30]. For these reasons, studies should always control for the influence of body size when investigating drivers of energy intake. However, not all of the variation in total energy intake is explained by differences in body size, with most studies leaving 45–55% of the variability unexplained [25,26,30,31]. Given our relatively limited ability to change homeostatic drivers of intake, interventions should focus on identifying behavioral contributors to this unexplained variability. Understanding the scope of cognitive contributors to food intake, beyond energy needs, is important for implementing effective appetite control strategies in people at risk for or with obesity.

The objective of the current study was to measure predictors of objective ad libitum energy intake at a post-exercise, laboratory-based test-meal in adolescents ages 10–16 years ranging in weight status from overweight to severe obesity. We investigated the contributions of both appetitive traits and states to post-exercise intake, independent of the effects of body size. We collected identical measures at a two-year follow-up visit to assess reliability and replicability of appetitive profiles and associations with post-exercise energy intake. We also tested whether baseline energy intake, appetitive states, and appetitive traits were associated with the change in adolescents’ weight and body fat over the two-year period. We hypothesized that appetitive states, rather than appetitive traits, would be the strongest predictor of energy intake at a post-exercise test-meal, after controlling for body size. We also hypothesized that baseline energy intake and appetitive traits would be positively associated with the change in weight and body fat over time.

## 2. Materials and Methods

### 2.1. Study Design

Study information was provided to potential participants at an orientation visit for a larger, prospective cohort study (NCT02784509) [32,33], during which parents signed informed consent and children signed written assent to participate in the longitudinal cohort study. A subsample of participants ages 10–16 years (inclusive) with a BMI ≥ the 85th age- and sex-specific percentile based on parent reported height and weight at initial phone screen were offered enrollment in this ancillary study (NCT03611296).

If interested, parents and children signed an additional informed consent and written assent, respectively, for the ancillary study procedures at their baseline clinic visit. During this initial clinic visit, participants underwent a fasting blood draw and assessment of vital signs, anthropometrics to verify BMI percentile, dual energy x-ray absorptiometry (DXA) for body composition, and self-reported pubertal development. Participants also received a brief introduction to the cycle ergometer test with exercise testing staff. After completion of screening procedures at the initial clinic visit, the following additional eligibility criteria were assessed: ability to understand instructions and complete all study procedures; meeting physical (height and weight) restrictions for the cycle ergometer; no anemia; no pregnancy; not consuming a restrictive diet; no food allergies or intolerances; willing to consume test-meal foods; no contraindications to exercise testing [34]; and no significant physical or mental disabilities.

Eligible participants returned 7–21 days later for an ancillary visit where cardiorespiratory fitness testing, questionnaires, behavioral tasks, and the ad libitum dinner test meal were completed. Recruitment continued until the target sample size of 30 participants was enrolled and completed study procedures. Families were contacted by phone once every 6 months for retention purposes. Year 2 follow-up visits for the parent longitudinal cohort study and this ancillary study were conducted within a ±6-month scheduling window depending on the family’s schedule and availability.

**Figure 1:**
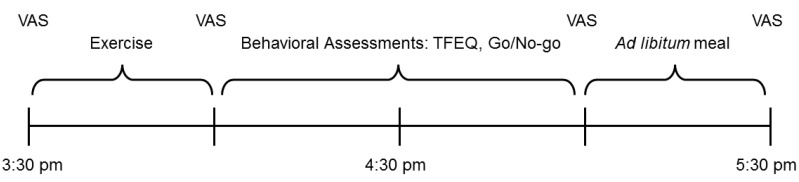
Timeline of study assessments. Visit procedures were identical at Baseline and Year 2. Abbreviations: visual analog scales, VAS; Three-Factor Eating Questionnaire, TFEQ.

Participants arrived at their clinic visits for the parent longitudinal study after an overnight fast (12 h), confirmed by parental report. The ancillary visits were conducted in the late afternoon (after school; between 3–4 p.m.) after a 3-h fast, confirmed by parental and self-report. The after-school timeframe was chosen to best accommodate family schedules, but also represents a time of day when youth this age would be most likely to engage in structured physical activity followed by an eating occasion. The timeline of study assessments is shown in Figure 1. The procedures described below were identical for Baseline and Year 2. All procedures were approved by the Institutional Review Board of Pennington Biomedical Research Center.

### 2.2. Measures

#### 2.2.1. Anthropometrics and Body Composition

Height (cm), weight (kg), and waist circumference (cm) were measured at the clinic visit by trained staff according to standard clinical procedures. These values were used to calculate BMI and age- and sex-specific BMI percentiles [35]. Adolescents with a BMI percentile ≥85th but <95th were considered overweight, and those with a BMI percentile ≥ 95th were considered to have obesity [35]. Severe obesity was defined as ≥120% of the 95th BMI percentile for age and sex. Body composition was measured with a General Electric whole-body iDXA scanner (GE Medical Systems, Milwaukee, WI, USA) while supine on the table in a hospital gown with all metal removed. Scans were automatically analyzed with Encore version 16.6 for Windows. Total fat mass (FM, kg) was extracted from the dataset and total FFM (kg) was calculated as body weight—FM. Fat-free mass index (FFMI; kg/m^2^) was calculated as FFM (kg) divided by height (m) squared and used as the indicator of body size [36].

#### 2.2.2. Exercise Testing

Cardiorespiratory fitness (VO2peak) was determined with a graded cycle ergometer test with the use of standard open-circuit metabolic cart (ParvoMedics, TrueOne 2400, Sandy, UT, USA) until volitional fatigue. This protocol was designed to measure steady-state responses to exercise at multiple submaximal stages leading up to a maximum workload [37]. Prior to beginning the test, participants underwent a 5-min warm up on the cycle ergometer to ensure comfortable positioning on the bike and practice pedaling at the appropriate cadence (60 revolutions per minute). Participants then commenced with an unloaded stage, and then the workload increased in 35 Watt increments until at least 2 submaximal (loaded) steady state measurements were obtained. These initial stages were 3 min in length. Closer to maximum, the workload increased in smaller, 15 Watt increments every minute so that there were no abrupt increases in workload, just a gradual rise in work intensity to fatigue. Heart rate was monitored by a Polar sensor (Polar Electro Inc., Bethpage, NY, USA) and Zephyr BioHarness (Medtronic, Boulder, CO, USA) worn around the chest, and blood pressure was taken manually with a sphygmomanometer. The test concluded with a monitored active cool-down phase.

#### 2.2.3. Trait Measures

Trait measures were assessed once at each time point, prior to the ad libitum test meal (i.e., in the fasted state). The measures described herein were selected to capture ingestive behavior constructs of interest in obesity and weight regulation, including disinhibition, cognitive restraint, emotional eating, and food reward. Participants completed the Three-Factor Eating Questionnaire revised 18-item version 2 (TFEQ-R18v2) with subscales for Uncontrolled Eating, Cognitive Restraint, and Emotional Eating [38,39]. This questionnaire is a shortened version of the original TFEQ or Eating Inventory [40], which is a common assessment of eating behaviors that has been well validated in the fields of obesity and ingestive behavior. This measure has previously been used in North American adolescent populations. Data for this survey were collected and managed using Research Electronic Data Capture (REDCap) tools [41,42].

To assess disinhibition more generally, in addition to food-specific disinhibition (i.e., uncontrolled eating), participants also completed a go/no-go inhibitory control task in E-Prime (version 2.0). The go/no-go tasks involve responding to a certain stimulus (e.g., button presses to zoo animal pictures) and refraining from responding to another stimulus (e.g., pictures of orangutans) [43]. This task measures response and motor inhibition. The task had 75% go trials and 25% no-go trials. Participants were instructed that they were helping the zookeeper round up animals that had escaped the zoo. They were instructed to press the spacebar when they saw an animal, which will capture that animal. However, orangutans were helping the zookeeper catch the other animals. Thus, participants were instructed to refrain from hitting the spacebar when they saw pictures of orangutans. Participants were asked to perform the task as accurately and quickly as possible. Reaction times and reaction accuracy were recorded and used to calculate error rates and false alarm rates. The task had a baseline version and a motivated version with three conditions: win a favorite snack, win a least favorite snack, and no prize. We also administered a motivated version, wherein a research assistant indicated prior to each run whether they were playing to win their favorite snack, least favorite snack, or no prize. They were told the prizes would be awarded at the end of the visit depending on how quickly and accurately they played the game. Both snacks were provided after the test-meal, regardless of task performance. Favorite and least favorite snacks were chosen from a list of 11 common and familiar snack items for this age range (e.g., chocolate, pretzels, celery) and the task was customized to each participant at each time point.

#### 2.2.4. State Measures

State measures were taken at multiple time points throughout each visit, including pre-exercise, post-exercise, pre-meal, and post-meal. The measures described herein were selected to capture subjective appetite and food wanting as potential drivers of food intake. For the purposes of this analysis, we only report the pre-meal appetitive state and explicit wanting outcomes as the most proximal to the initiation of the ad libitum test-meal.

We assessed appetitive states with standard 100 mm visual analog scales (VAS): hunger, fullness, desire to eat, prospective food consumption, and satisfaction [16]. These scales were selected as they have been well validated and are commonly used to quantify appetite. Participants were instructed, “You will answer several questions about feelings such as hunger and fullness. Be sure to read the question and the ends of the scale carefully and think about how you feel at this moment. Then move the cursor to the point on the scale that best describes how you are feeling. Please let a researcher know if you have any questions”.

In addition to overall appetite ratings, explicit wanting for specific foods (including those served at the meal) was also assessed by VAS. Participants were instructed to look at each food image and then indicate whether or not they are familiar with that food. If yes, they were asked to rate how much they want to eat that food on a sliding scale with anchors marked “not at all” on the left and “want very much” on the right. Average explicit wanting was calculated for each time point. Data were collected and managed using REDCap.

#### 2.2.5. Ad Libitum Test-Meal

Objective energy intake (kcal) was measured at an ad libitum, multi-item dinner test-meal that occurred approximately one hour after the exercise test. Participants were offered standardized portions of common and familiar foods for this age group, including macaroni and cheese, chicken nuggets, broccoli with butter, and chocolate chip cookies with ketchup and barbeque sauce for dipping and water to drink (see Supplementary Table S1). Foods were prepared by a metabolic kitchen and offered in excess of energy needs totaling ~3000 kcal across all items. Participants were instructed, “This meal is to serve as your evening meal. During this meal, you may eat as much or as little of these foods as you would like. While you are eating, please do not use your phone. When you have finished, you can use the intercom to let me know that you are done. Do you have any questions?” Pre- and post-meal weights (grams) were taken for each food item, with the different in weight representing the amount of food eaten. Gram weights were converted to energy intake (kcal) and nutrient intakes using nutrition facts information databases.

### 2.3. Statistical Analysis

The following statistical approaches were applied to data collected at Baseline and Year 2, separately. Energy intake (kcal) was adjusted for FFMI by taking the unstandardized residual of the linear regression between the two variables. Pearson’s correlations were used to test associations between energy intake (adjusted for FFMI) with trait variables (TFEQ uncontrolled eating, emotional eating, cognitive restraint; go/no-go errors, false alarms) and state variables (pre-meal VAS hunger, fullness, desire to eat, prospective food consumption, satisfaction; pre-meal explicit wanting for foods). Significant correlates were entered into a stepwise linear regression model to determine the strongest profile that would predict energy intake (adjusted for FFMI) as the dependent variable, controlling for age and sex where appropriate. Retention of covariates was determined by stepwise linear regression, and age and sex were non-significant and therefore not included in any final models.

Intra-class correlation coefficients were computed using the two-way mixed procedure with absolute agreement. One-sample t-tests were used to examine changes in energy intake, body weight, and fat mass from Baseline to Year 2. In longitudinal analyses, linear regression models were used to test the effects of baseline energy intake at the post-exercise test-meal (adjusted for FFMI) on the change in weight and fat mass from Baseline to Year 2. Similarly, we tested the change in energy intake (adjusted for FFMI) from Baseline to Year 2 as the independent variable. Analyses were conducted in SPSS (version 25, IBM SPSS Statistics) and results were considered significant at *p* < 0.05.

## 3. Results

### 3.1. Participant Characteristics

Characteristics of the study sample are described in Table 1. Thirty adolescents (mean age 12.4 ± 1.9 years, 50% female) were enrolled in the study, with a weight status distribution across the following weight categories: overweight (20%); obesity, not severe (43%); and severe obesity (37%). At Year 2, nineteen participants (mean age 14.6 ± 2.0 years, 47% female) remained in the study, with a weight status distribution across healthy weight (10%); overweight (11%); obesity, not severe (42%); and severe obesity (37%). In the time between Baseline and Year 2, five participants were lost to follow-up, two relocated, two changed their minds regarding participation, and two were excluded due to safety concerns (i.e., contraindications to exercise testing) by the study investigators. Children who remained in the study did not differ from dropouts in terms of their age (*p* = 0.61), sex (*p* = 0.71), or weight status (*p* = 0.62) at baseline.

An average of 22.2 ± 3.7 months passed between visits. Four participants completed follow-up assessments in the summer of 2020, following the end of local stay-at-home orders and clinical research suspension at the start of the COVID-19 pandemic. While the time between visits for these four participants was significantly longer compared to the rest of the study sample (28.5 vs. 20.5 months, respectively; t = 7.14, *p* = 0.002), they did not differ from the other participants in terms of change in weight, fat mass, or energy intake over time (all *p* > 0.28), nor was the time between visits associated with any of these outcome measures (all *p* > 0.11). Therefore, all analyses were conducted using the full sample.

Participants experienced a significant increase in body weight over the two-year period (t = 6.65, *p* < 0.001). In this same time frame, they showed a significant increase in fat mass (t = 4.18, *p* = 0.001) and in FFMI (t = 10.6, *p* < 0.001). From Baseline to Year 2, there were no significant changes in test-meal energy intake (absolute t = 0.34, *p* = 0.74; adj. for FFMI t = 1.53, *p* = 0.14).

### 3.2. Energy Intake Reliability

**Figure 2:**
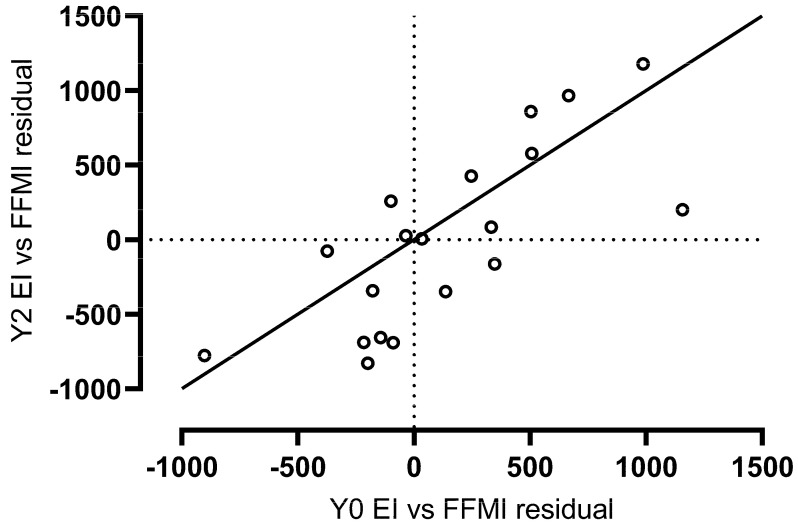
Association between energy intake (adj. for FFMI) at Baseline and Year 2 (ICC = 0.84), depicted against a line of identity (solid line). Abbreviations: energy intake, EI; fat-free mass index, FFMI; intra-class correlation, ICC; baseline, Y0; year 2, Y2.

The intra-class correlation for energy intake (adj. for FFMI) at Baseline and Year 2 (*n* = 19) was 0.84, indicating good reliability over time. The correlation between Baseline and Year 2 is depicted in Figure 2.

### 3.3. Baseline Predictors of Energy Intake (n = 30)

Correlations are presented in Table 2. Pre-meal prospective food consumption (r = 0.50), pre-meal hunger (r = 0.39), and TFEQ Uncontrolled Eating (r = 0.40) were positively associated with post-exercise test-meal energy intake (adj. for FFMI) (all *p* < 0.05). Pre-meal fullness (r = −0.42, *p* = 0.02) was negatively associated with test-meal energy intake (adj. for FFMI). There was a trend for pre-meal food wanting to be associated with test-meal energy intake (r = 0.35, *p* = 0.06). None of the go/no-go task outcomes (false alarms, error rates) were associated with test-meal energy intake, regardless of whether it was a food-motivated (win a favorite or least favorite snack) or neutral condition (all *p* > 0.23). In stepwise linear regression, pre-meal prospective food consumption was the strongest and sole predictor of test-meal energy intake (β = 11.1, *p* = 0.005) included in the model (R^2^ = 0.25, *p* = 0.005), regardless of age (*p* = 0.69) and sex (*p* = 0.37).

### 3.4. Year 2 Follow-Up Predictors of Energy Intake (n = 19)

Correlations are presented in Table 2. At Year 2, all of the pre-meal appetite scores were significantly associated with post-exercise test-meal energy intake (adj. for FFMI). Pre-meal prospective food consumption (r = 0.64), desire to eat (r = 0.61), and hunger (r = 0.55) were all positively associated with test-meal energy intake, while pre-meal satisfaction (r = −0.59) and fullness (r = −0.47) were both negatively associated with test-meal energy intake (all *p* < 0.05). Pre-meal food wanting was not associated with test-meal energy intake (*p* = 0.75). None of the TFEQ subscale scores, nor the go/no-go task outcomes, were associated with energy intake (all *p* > 0.27). In stepwise linear regression, pre-meal prospective food consumption was the strongest and sole predictor of test-meal energy intake (adj. for FFMI) (β = 22.2, *p* = 0.003) in the final model (R^2^ = 0.41, *p* = 0.003).

### 3.5. Associations with Weight and Fat Mass Change over Two Years

**Figure 3:**
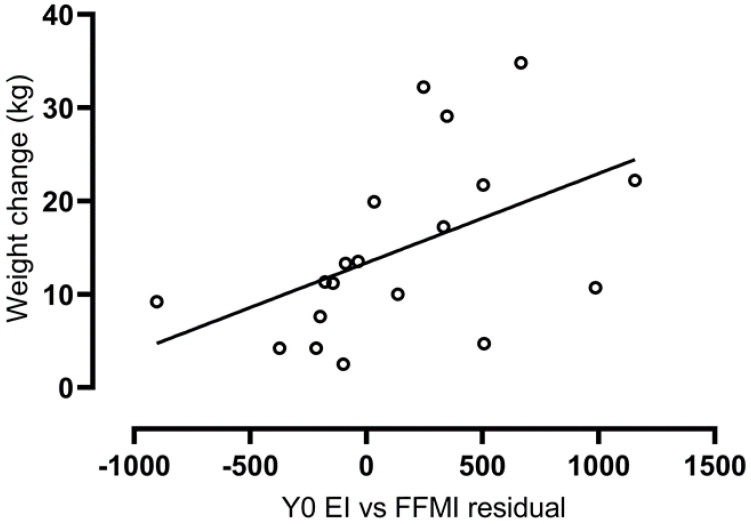
Association between energy intake (adj. for FFMI) at Baseline and weight change (kg) from Baseline to Year 2 (r = 0.49, *p* = 0.04). Abbreviations: energy intake, EI; fat-free mass index, FFMI; baseline, Y0.

Baseline test-meal energy intake (adj. for FFMI) (β = 0.01, *p* = 0.04) was positively associated with the change in body weight (kg) (R^2^ = 0.24, *p* = 0.04; Figure 3). However, baseline test-meal energy intake (adj. for FFMI) was not associated with the change in fat mass (kg) (R^2^ = 0.14, *p* = 0.11). None of the other baseline appetitive states or traits were associated with the change in body weight or fat mass (all *p* > 0.22).

The change in test-meal energy intake (adj. for FFMI) from Baseline to Year 2 was not significantly associated with the change in body weight (*p* = 0.17), nor with the change in fat mass (*p* = 0.22) over the two-year period.

## 4. Discussion

The present study aimed to assess drivers of post-exercise energy intake among adolescents ranging in weight status from overweight to severe obesity. The longitudinal design allowed us to test associations between eating behavior profiles and weight change over two years. Independent of the influence of body size, our results suggest that food intake at the post-exercise meal was more strongly associated with current appetitive state than with eating behavior traits, in line with our hypothesis. In adolescents with overweight to severe obesity, the amount of food that participants thought they could eat (i.e., prospective food consumption) was positively associated with actual consumption, regardless of body size. This pattern of association was replicated at the two-year follow-up. Baseline post-exercise energy intake (adj. for FFMI) was associated with the change in weight over two years. However, contrary to our hypothesis, baseline appetitive traits were not associated with change in weight or body fat.

Test-meal energy intake adjusted for body size was consistent over the two-year period, with a strong correlation between residuals from Baseline and Year 2. This suggests that the homeostatic drive is retained despite the presence of obesity and the effects of growth and weight gain. At Baseline, approximately 25% of the variability in energy intake (controlling for body size) was explained by prospective food consumption. This increased to 41% of the variability at Year 2. Prospective food consumption is a subjective rating of the amount of food a person thinks they can eat at a particular moment. In this study, we used VAS ratings taken approximately 1 h after exercise, immediately before the test-meal. Participants with a higher rating of pre-meal prospective food consumption did consume more calories at the subsequent meal, suggesting an intentional awareness regarding the amount or portion size they planned to eat. This is counter to previous reports in people with obesity that intake of an objectively large amount of food is often driven by a loss of control over intake during an eating occasion [6,14,44]. Though we saw a signal for uncontrolled eating (TFEQ) being associated with energy intake at Baseline, this was no longer significant in the regression model with other appetite variables, nor was there an association at Year 2. Other VAS appetitive state measures were correlated with energy intake, including pre-meal hunger and fullness, but were not retained in the model.

Despite weight gain over the course of the two-year period, these eating behavior profiles were preserved. In other words, weight gain over time did not result in a reduction in eating behaviors (i.e., high prospective food consumption, greater energy intake) that predispose individuals to additional weight gain. Adolescents who ate more food in relation to their body size continued to do so two years later. Studies in adults have posited that weight gain propensity should be diminished once weight gain has occurred, at least from a metabolic perspective [45]. However, it is unclear whether this principle would apply to behavioral risk factors for obesity or to adolescent populations still experiencing periods of accelerated growth. In this sample, participants experienced an average rate of weight gain of approximately 7 kg per year, slightly more than the typical weight gain of 4 to 5 kg per year in adolescents with obesity [46]. About half of this weight gain was fat mass. Our preliminary results do not show any indication of a natural reduction in the propensity for weight gain over time, suggesting that behavioral intervention would be beneficial to reduce excess energy intake.

Contrary to previous studies, we did not find an association between appetitive traits (i.e., disinhibition, TFEQ) and weight gain over time. This could be due to the fact that we used versions of these assessments that were adapted for children and adolescents and therefore may have captured slight differences in cognitive constructs of interest. We used a food motivation manipulation in the go/no-go task, but performance (i.e., error and false alarm rates) across trials was not affected by the presence of food rewards (data not shown). For this reason, we treated the go/no-go task as a trait measure of disinhibition. Go/no-go task performance was not associated with energy intake, eating behaviors, or weight gain in this study sample. The revised 18-item version of the TFEQ that we used has only one overlapping subscale (cognitive restraint) with the original version developed by Stunkard and Messick [40]. Uncontrolled eating captures some similar behaviors to the original disinhibition subscale, but its application in the ingestive behavior and obesity literature is relatively limited. Studies that have used the TFEQ-R18v2 have found a negative association between cognitive restraint and BMI, while uncontrolled eating and emotional eating were positively associated with BMI [39,47].

Strengths of the current study include the balanced racial and ethnic diversity of the study sample, as well as the representation of adolescents ranging in weight status from overweight to severe obesity. In addition, we assessed several state and trait eating behavior constructs in relation to an objective ad libitum test-meal after controlled exercise. Limitations of the current study include the small sample size and loss of participants at the Year 2 follow-up. However, the reliability of our eating behavior assessments and replication of correlations increases our confidence in the effects seen. Future larger studies could examine potential differences in post-exercise eating behaviors by race/ethnicity, age, or sex. We were unable to test the direct impact of reproductive hormones and physiological maturation on our study outcomes. However, self-reported Tanner stages collected for this study were strongly associated with both age and FFMI (data not shown), which we did account for in our analysis. A future study designed specifically to address the impact of the pubertal transition on eating behaviors and weight gain would be of interest. Finally, we also lacked a non-exercise control condition, and the exercise itself was not standardized in terms of duration nor energy expenditure. The current study also did not examine multiple exercise intensities. Future studies could examine differences in predictors of energy intake between various exercise and non-exercise conditions. This line of work could further support the hypothesis that exercise sensitizes individuals to appetitive state and determine the optimal exercise parameters (e.g., intensity, modality, caloric expenditure) needed for such an effect [48]. Additional future work could examine the potential role of appetite-regulating hormones that are released in response to exercise [49,50] in the relationship between subjective appetite and subsequent energy intake in this population.

## 5. Conclusions

In sum, we found that appetitive state was the strongest predictor of post-exercise energy intake and that energy intake at Baseline predicted two-year weight gain. Future studies could examine whether post-exercise prospective food consumption is modifiable through cognitive behavioral strategies, with a goal to reduce compensatory food intake and risk for weight gain over time.

## Figures and Tables

**Table 1:** Participant characteristics at Baseline and Year 2 follow-up and change over time. Presented as mean (SD) or *n* (%). Abbreviations: Three Factor Eating Questionnaire Revised 18-item version 2, TFEQ-R18v2.

	Baseline (*n* = 30)		Year 2 (*n* = 19)		Change
Sex					
Male	15	(50%)	10	(53%)	
Female	15	(50%)	9	(47%)	
Race/ethnicity					
Black or African-American	14	(47%)	12	(63%)	
Non-Hispanic White	13	(43%)	6	(32%)	
Hispanic	3	(10%)	1	(5%)	
Weight status					
Healthy weight	0		2	(11%)	
Overweight	5	(17%)	2	(11%)	
Obesity, not severe	14	(47%)	8	(42%)	
Obesity, severe	11	(37%)	7	(37%	
Age (year)	12.9	(1.9)	14.6	(2.0)	1.7
Weight (kg)	78.7	(20.5)	91.8	(24.5)	13.1
Fat mass (kg)	32.4	(11.5)	38.4	(16.9)	6.0
Fat-free mass index (kg/m^2^)	16.7	(2.5)	19.0	(2.7)	2.3
Test-meal energy intake (kcal)	1078	(524)	1252	(610)	174
TFEQ-R18v2					
Uncontrolled Eating	34	(22)	38	(18)	4
Cognitive Restraint	36	(22)	31	(26)	−5
Emotional Eating	24	(23)	24	(22)	0

**Table 2:** Pearson’s correlations between state or trait measures and energy intake (adj. for FFMI) at Baseline and Year 2 follow-up. Abbreviations: Three Factor Eating Questionnaire Revised 18-item version 2, TFEQ-R18v2; VAS, visual analog scales. * *p* < 0.05, ** *p* < 0.01.

	Baseline Energy Intake (*n* = 30)	Year 2 Energy Intake (*n* = 19)
Trait Measures		
TFEQ-R18v2		
Uncontrolled Eating	0.40 *	0.22
Cognitive Restraint	0.04	−0.02
Emotional Eating	0.27	−0.12
Go/no-go Task		
False alarms (%)	0.23	0.27
Error rates (%)	0.13	0.26
State Measures		
Pre-meal VAS		
Hunger	0.39 *	0.55 *
Fullness	−0.42 *	−0.47 *
Desire to eat	0.26	0.61 **
Prospective food consumption	0.50 **	0.64 **
Satisfaction	−0.31	−0.59 **
Pre-meal explicit wanting	0.35	0.08

## Data Availability

Data are available from the corresponding author upon reasonable request and data transfer/use agreement, per standard institutional procedures.

## Supplementary Materials

The following are available online at https://www.mdpi.com/article/10.3390/nu14010223/s1, Table S1: Menu and portion sizes of food served at the post-exercise ad libitum dinner test-meal.

## References

[1] Keller K.L., Kling S.M.R., Fuchs B., Pearce A.L., Reigh N.A., Masterson T., Hickok K. (2019). A Biopsychosocial Model of Sex Differences in Children’s Eating Behaviors. Nutrients.

[2] Russell C.G., Russell A. (2019). A biopsychosocial approach to processes and pathways in the development of overweight and obesity in childhood: Insights from developmental theory and research. Obes. Rev..

[3] Kininmonth A., Smith A., Carnell S., Steinsbekk S., Fildes A., Llewellyn C. (2021). The association between childhood adiposity and appetite assessed using the Child Eating Behavior Questionnaire and Baby Eating Behavior Questionnaire: A systematic review and meta-analysis. Obes. Rev..

[4] Batterink L., Yokum S., Stice E. (2010). Body mass correlates inversely with inhibitory control in response to food among ad-olescent girls: An fMRI study. Neuroimage.

[5] Price M., Lee M., Higgs S. (2016). Food-specific response inhibition, dietary restraint and snack intake in lean and over-weight/obese adults: A moderated-mediation model. Int. J. Obes..

[6] Vainik U., Garcia I.G., Dagher A. (2019). Uncontrolled eating: A unifying heritable trait linked with obesity, overeating, personality and the brain. Eur. J. Neurosci..

[7] Brunner E.J., Maruyama K., Shipley M., Cable N., Iso H., Hiyoshi A., Stallone D., Kumari M., Tabak A., Singh-Manoux A. (2021). Appetite disinhibition rather than hunger explains genetic effects on adult BMI trajectory. Int. J. Obes..

[8] Garcia-Garcia I., Neseliler S., Morys F., Dadar M., Yau Y.H.C., Scala S.G., Zeighami Y., Sun N., Collins D.L., Vainik U. (2021). Relationship between impulsivity, uncontrolled eating and body mass index: A hierarchical model. Int. J. Obes..

[9] de Lauzon-Guillain B., Basdevant A., Romon M., Karlsson J., Borys J.M., Charles M.A. (2006). FLVS Study Group Is restrained eating a risk factor for weight gain in a general population?. Am. J. Clin. Nutr..

[10] Appelhans B.M., Woolf K., Pagoto S., Schneider K.L., Whited M.C., Liebman R. (2011). Inhibiting Food Reward: Delay Discounting, Food Reward Sensitivity, and Palatable Food Intake in Overweight and Obese Women. Obesity.

[11] Epstein L.H., Yokum S., Feda D.M., Stice E. (2014). Food reinforcement and parental obesity predict future weight gain in non-obese adolescents. Appetite.

[12] Rollins B.Y., Loken E., Savage J.S., Birch L.L. (2014). Measurement of food reinforcement in preschool children. Associations with food intake, BMI, and reward sensitivity. Appetite.

[13] French S.A., Epstein L.H., Jeffery R.W., Blundell J.E., Wardle J. (2012). Eating behavior dimensions. Associations with energy intake and body weight. A review. Appetite.

[14] Feig E.H., Piers A.D., Kral T.V., Lowe M.R. (2018). Eating in the absence of hunger is related to loss-of-control eating, hedonic hunger, and short-term weight gain in normal-weight women. Appetite.

[15] Sadoul B.C., Schuring E.A., Mela D.J., Peters H.P. (2014). The relationship between appetite scores and subsequent energy intake: An analysis based on 23 ran-domized controlled studies. Appetite.

[16] Stubbs R.J., Hughes D.A., Johnstone A.M., Rowley E., Reid C., Elia M., Stratton R., Delargy H., King N., Blundell J.E. (2000). The use of visual analogue scales to assess motivation to eat in human subjects: A review of their reliabil-ity and validity with an evaluation of new hand-held computerized systems for temporal tracking of appetite ratings. Br. J. Nutr..

[17] Ruddick-Collins L.C., Byrne N., King N.A. (2018). Assessing the influence of fasted and postprandial states on day-to-day variability of appetite and food preferences. Physiol. Behav..

[18] Flint A., Raben A., Blundell J.E., Astrup A. (2000). Reproducibility, power and validity of visual analogue scales in assessment of appetite sensations in single test meal studies. Int. J. Obes..

[19] Smethers A.D., Roe L.S., Sanchez C.E., Zuraikat F.M., Keller K.L., Kling S.M.R., Rolls B.J. (2019). Portion size has sustained effects over 5 days in preschool children: A randomized trial. Am. J. Clin. Nutr..

[20] Kling S.M., Roe L.S., Keller K.L., Rolls B.J. (2016). Double trouble: Portion size and energy density combine to increase preschool children’s lunch intake. Physiol. Behav..

[21] Boutelle K.N., Manzano M.A., Eichen D.M. (2020). Appetitive traits as targets for weight loss: The role of food cue respon-siveness and satiety responsiveness. Physiol. Behav..

[22] Dalton M., Hollingworth S., Blundell J., Finlayson G. (2015). Weak Satiety Responsiveness Is a Reliable Trait Associated with Hedonic Risk Factors for Overeating among Women. Nutrients.

[23] Edholm O.G. (1956). Energy expenditure in relation to nutrition. Proc. Nutr. Soc..

[24] Mayer J., Roy P., Mitra K.P. (1956). Relation between caloric intake, body weight, and physical work: Studies in an industrial male population in West Bengal. Am. J. Clin. Nutr..

[25] Blundell J.E., Caudwell P., Gibbons C., Hopkins M., Näslund E., King N.A., Finlayson G. (2012). Body composition and appetite: Fat-free mass (but not fat mass or BMI) is positively associated with self-determined meal size and daily energy intake in humans. Br. J. Nutr..

[26] Caudwell P., Finlayson G., Gibbons C., Hopkins M., King N., Näslund E., Blundell J.E. (2012). Resting metabolic rate is associated with hunger, self-determined meal size, and daily energy intake and may represent a marker for appetite. Am. J. Clin. Nutr..

[27] Weise C.M., Hohenadel M.G., Krakoff J., Votruba S.B. (2013). Body composition and energy expenditure predict ad-libitum food and macronutrient intake in humans. Int. J. Obes..

[28] Hopkins M., Finlayson G., Duarte C., Whybrow S., Ritz P., Horgan G.W., Blundell J.E., Stubbs R.J. (2015). Modelling the associations between fat-free mass, resting metabolic rate and energy intake in the con-text of total energy balance. Int. J. Obes..

[29] Cameron J.D., Sigal R.J., Kenny G.P., Alberga A.S., Prud’Homme D., Phillips P., Doucette S., Goldfield G. (2016). Body composition and energy intake—Skeletal muscle mass is the strongest predictor of food intake in obese adolescents: The HEARTY trial. Appl. Physiol. Nutr. Metab..

[30] Fearnbach S.N., Masterson T.D., Schlechter H.A., Loken E., Downs D.S., Thivel D., Keller K.L. (2017). Perceived Exertion during Exercise Is Associated with Children’s Energy Intake. Med. Sci. Sports Exerc..

[31] Hopkins M., Finlayson G., Duarte C., Gibbons C., Johnstone A., Whybrow S., Horgan G.W., Blundell J.E., Stubbs R.J. (2018). Biological and psychological mediators of the relationships between fat mass, fat-free mass and energy intake. Int. J. Obes..

[32] Kracht C., Champagne C.M., Hsia D.S., Martin C.K., Newton R.L., Katzmarzyk P.T., Staiano A.E. (2020). Association Between Meeting Physical Activity, Sleep, and Dietary Guidelines and Cardiometabolic Risk Factors and Adiposity in Adolescents. J. Adolesc. Health.

[33] Kracht C.L., Chaput J.-P., Martin C.K., Champagne C.M., Katzmarzyk P.T., Staiano A.E. (2019). Associations of Sleep with Food Cravings, Diet, and Obesity in Adolescence. Nutrients.

[34] American College of Sports Medicine (2018). ACSM’s Guidelines for Exercise Testing and Prescription.

[35] Kuczmarski R., Ogden C., Guo S., Grummer-Strawn L.M., Flegal K., Mei Z., Wei R., Curtin L., Roche A., Johnson C. (2002). 2000 CDC Growth Charts for the United States: Methods and Development.

[36] VanItallie T.B., Yang M.U., Heymsfield S.B., Funk R.C., Boileau R.A. (1990). Height-normalized indices of the body’s fat-free mass and fat mass: Potentially useful indicators of nutritional status. Am. J. Clin. Nutr..

[37] Fearnbach S.N., Johannsen N.M., Martin C.K., Katzmarzyk P.T., Beyl R.A., Hsia D., Carmichael O.T., Staiano A.E. (2020). A Pilot Study of Cardiorespiratory Fitness, Adiposity, and Cardiometabolic Health in Youth with Overweight and Obesity. Pediatr. Exerc. Sci..

[38] Karlsson J., Persson L.-O., Sjöström L., Sullivan M. (2000). Psychometric properties and factor structure of the Three-Factor Eating Questionnaire (TFEQ) in obese men and women. Results from the Swedish Obese Subjects (SOS) study. Int. J. Obes..

[39] Cappelleri J.C., Bushmakin A.G., Gerber R.A., Leidy N.K., Sexton C.C., Lowe M.R., Karlsson J. (2009). Psychometric analysis of the Three-Factor Eating Questionnaire-R21: Results from a large diverse sample of obese and non-obese participants. Int. J. Obes..

[40] Stunkard A.J., Messick S. (1985). The three-factor eating questionnaire to measure dietary restraint, disinhibition and hunger. J. Psychosom. Res..

[41] Harris P.A., Taylor R., Thielke R., Payne J., Gonzalez N., Conde J.G. (2009). Research electronic data capture (REDCap)—A metadata-driven methodology and workflow process for providing translational research informatics support. J. Biomed. Inform..

[42] Harris P.A., Taylor R., Minor B.L., Elliott V., Fernandez M., O’Neal L., McLeod L., Delacqua G., Delacqua F., Kirby J. (2019). The REDCap consortium: Building an international community of software platform partners. J. Biomed. Inform..

[43] Grammer J.K., Carrasco M., Gehring W.J., Morrison F.J. (2014). Age-related changes in error processing in young children: A school-based investigation. Dev. Cogn. Neurosci..

[44] Tanofsky-Kraff M., Yanovski J., Ba N.A.S., Olsen C.H., Bs J.G., Yanovski J.A. (2009). A prospective study of loss of control eating for body weight gain in children at high risk for adult obesity. Int. J. Eat. Disord..

[45] Ravussin E. (1993). Energy Metabolism in Obesity: Studies in the Pima Indians. Diabetes Care.

[46] Lange S.J., Kompaniyets L., Freedman D.S., Kraus E.M., Porter R., Blanck H.M., Goodman A.B. (2021). Dnp3 Longitudinal Trends in Body Mass Index before and during the COVID-19 Pandemic among Persons Aged 2–19 Years—United States, 2018–2020. MMWR Morb. Mortal. Wkly. Rep..

[47] Calvo D., Galioto R., Gunstad J., Spitznagel M.B. (2014). Uncontrolled eating is associated with reduced executive functioning. Clin. Obes..

[48] Thivel D., Rumbold P., King N.A., Pereira B., Blundell J.E., Mathieu M.-E. (2016). Acute post-exercise energy and macronutrient intake in lean and obese youth: A systematic review and meta-analysis. Int. J. Obes..

[49] Hazell T.J., Islam H., Townsend L.K., Schmale M.S., Copeland J.L. (2016). Effects of exercise intensity on plasma concentrations of appetite-regulating hormones: Potential mechanisms. Appetite.

[50] Ouerghi N., Feki M., Bragazzi N.L., Knechtle B., Hill L., Nikolaidis P.T., Bouassida A. (2021). Ghrelin Response to Acute and Chronic Exercise: Insights and Implications from a Systematic Review of the Literature. Sports Med..

