# Supplementary material for: Predictors of Post-Exercise Energy Intake in Adolescents Ranging in Weight Status from Overweight to Severe Obesity
Source: Nutrients. 2022 Jan 5;14(1):223. doi: 10.3390/nu14010223 (PMC8747392; doi:10.3390/nu14010223)
Supplement: Supplementary file 1 [file nutrients-14-00223-s001.zip › nutrients-1496258-supplementary.pdf]

Supplemental Table S1. Menu and portion sizes of food served at the post-exercise *ad libitum* dinner test-meal. No additional servings were made available. Macronutrient distribution (% of total kcal) was 44.4% carbohydrate, 40.9% fat, and 14.7% protein.

**Food Item**	**Food weight (g)**	**Energy (kcal)**
Chicken nuggets (Applause)	356	801
Macaroni pasta with cheese (Stouffer's)	600	888
Chocolate chip cookies (Famous Amos)	150	776
Steamed broccoli with butter (Sysco, Wholesome Farms)	320	227
Ketchup (Heinz)	108	120
Barbeque sauce (Heinz)	112	140
Water (32 fl oz)	946	0
TOTAL	1,646	2,952
